# Influence of the shape and surface oxidation in the magnetization reversal of thin iron nanowires grown by focused electron beam induced deposition

**DOI:** 10.3762/bjnano.6.136

**Published:** 2015-06-15

**Authors:** Luis A Rodríguez, Lorenz Deen, Rosa Córdoba, César Magén, Etienne Snoeck, Bert Koopmans, José M De Teresa

**Affiliations:** 1 Laboratorio de Microscopias Avanzadas (LMA) - Instituto de Nanociencia de Aragón (INA), Universidad de Zaragoza, 50018 Zaragoza, Spain; 2 Departamento de Física de la Materia Condensada, Universidad de Zaragoza, 50009 Zaragoza, Spain; 3 CEMES-CNRS 29, rue Jeanne Marvig, B.P. 94347, F-31055 Toulouse Cedex, France; 4 Department of Applied Physics, Eindhoven University of Technology, PO Box 513, 5600 MB Eindhoven, Netherlands; 5 Fundación ARAID, 50018 Zaragoza, Spain; 6 Instituto de Ciencia de Materiales de Aragón (ICMA), Universidad de Zaragoza-CSIC, 50009 Zaragoza, Spain

**Keywords:** coercive field, focused electron beam induced deposition, iron nanowires, magnetization reversal, magneto-optical Kerr effect, transmission electron microscopy

## Abstract

Iron nanostructures grown by focused electron beam induced deposition (FEBID) are promising for applications in magnetic sensing, storage and logic. Such applications require a precise design and determination of the coercive field (*H*_C_), which depends on the shape of the nanostructure. In the present work, we have used the Fe_2_(CO)_9_ precursor to grow iron nanowires by FEBID in the thickness range from 10 to 45 nm and width range from 50 to 500 nm. These nanowires exhibit an Fe content between 80 and 85%, thus giving a high ferromagnetic signal. Magneto-optical Kerr characterization indicates that *H*_C_ decreases for increasing thickness and width, providing a route to control the magnetization reversal field through the modification of the nanowire dimensions. Transmission electron microscopy experiments indicate that these wires have a bell-type shape with a surface oxide layer of about 5 nm. Such features are decisive in the actual value of *H*_C_ as micromagnetic simulations demonstrate. These results will help to make appropriate designs of magnetic nanowires grown by FEBID.

## Introduction

The fabrication of magnetic nanostructures in a single lithographic step by focused electron beam induced deposition (FEBID) is currently an exciting research topic [1–2]. In this technique, a scanning electron microscope (SEM) dissociates the precursor molecules delivered into the area of interest by a gas-injection system, producing a deposit [3–6]. The use of precursor molecules containing cobalt [7–10] or iron [11–14] allows for the growth of magnetic nanostructures with tailored dimensions. Some of the most recent advances in this topic are: the achievement of resolution in single magnetic structures below 30 nm [15–17], the fabrication of nanomagnets for logic [18], the production of highly-dense isolated magnetic structures [19], the growth of three-dimensional nanowires [20–21] and the fabrication of nanospheres on scanning probe tips [22–23].

One of the crucial parameters to be controlled in such magnetic nanostructures grown by FEBID is the coercive field, *H*_C_, which corresponds to the magnetic field producing the magnetization reversal. Most of magnetic devices work by producing a voltage output when the magnetization reversal occurs. In the case of cobalt deposits, it was previously found that the coercive field is governed by shape anisotropy [24] due to the polycrystalline microstructure [25], and is thus a function of the deposit dimensions [26]. However, more detailed studies subsequently emphasized the role played by the halo and the effective magnetic shape in the coercive field of cobalt nanowires [27–29]. In brief, the halo structure around the main deposit (caused by proximity effects in FEBID [30]) is an easy place for nucleation of domain walls (DWs) [31] starting the magnetization reversal of the nanowire, which gives rise to low coercive fields. This can be a source of troubles if one naively aims to control the coercive field of magnetic nanostructures by means of the shape of the main deposit without taking into account the effect of the halo.

Regarding the case of iron nanostructures grown by FEBID, the work by Gavagnin et al. has highlighted that the coercive field could be controllable by means of the deposit thickness [18]. These authors found that nanomagnets with thicknesses of 25 and 35 nm (but equal length and width) produced different coercive fields [18]. However, a detailed explanation for such phenomenology was not provided. The same group later found that, similar to the case of cobalt nanowires grown by FEBID, a magnetic halo in iron nanowires is an easy nucleation center for domain walls, decreasing the observed coercive field [31].

At this point, further research towards a deeper understanding of the processes that determine the coercive field in magnetic nanostructures grown by FEBID is required. A true control over coercivity is the only way to design and fabricate appropriate magnetic devices based on FEBID materials. In the present work, we focus our attention on iron nanowires with fixed length (4 μm) and varying width (50–500 nm) and nominal thickness (*t*_Nom_) between 10 and 45 nm. We have chosen this geometry given our previous experience with cobalt nanowires grown by FEBID [24,27]. In these experiments mono-domain magnetic structures in remanence, as required in most applications, were obtained for nanowire widths around 400 nm or smaller and length/width aspect ratios of the order of 10. By performing systematic studies of the coercive field as a function of dimensions and carrying out micromagnetic simulations, we are able to conclude that the specific shape of the nanowire as well as the surface oxidation are key to explain the observed behavior. These will determine the effective magnetic shape of the nanowires, which will control the coercivity. This will be still more relevant as the dimensions of the nanowires become smaller, as it is desired in some applications where high integration of magnetic nanostructures is needed.

## Results and Discussion

### Growth of the nanowires

**Figure 1:**
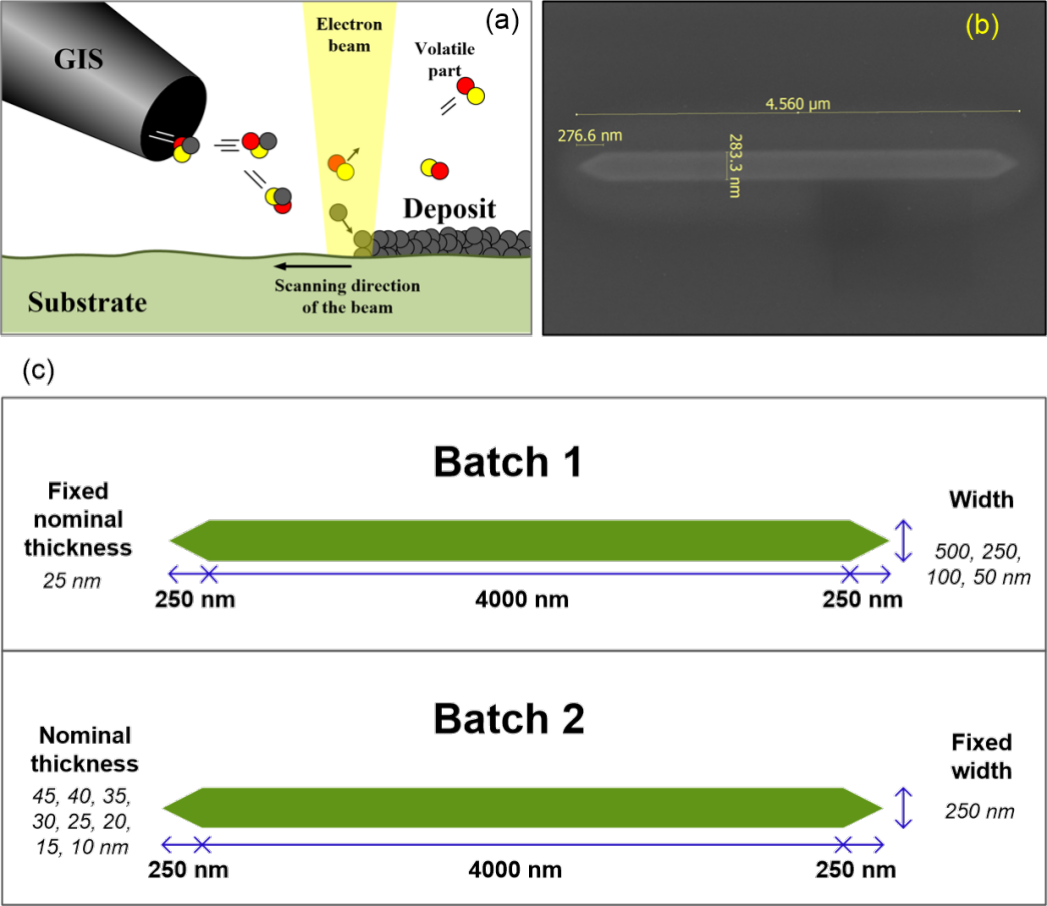
(a) A sketch of the FEBID process. (b) A SEM image of the nanowire with targeted width of 250 nm and nominal thickness of 15 nm. (c) Sketch of the two batches of nanowires: Batch 1 has got 25 nm nominal thickness and varying width whereas batch 2 has got 250 nm width and varying thickness.

The iron nanowires have been grown on B-doped Si substrates inside an FEI Helios 600 apparatus, using Fe_2_(CO)_9_ as precursor and the scanning electron microscope (SEM) to produce magnetic deposits in a single step, as sketched in Figure 1a. The precursor is delivered to the area of interest through a single gas-injection-system (GIS) with inner diameter of 160 μm, whose tip is located approximately 150 μm above (*z* direction) and 50 μm off (*x* direction) the central irradiation point of the electron beam. The base pressure inside the chamber (before the flow of the precursor gas) is about 1 × 10^−6^ mbar. The precursor flux can be controlled through a leak valve in the GIS and had to be optimized in order to obtain deposits exhibiting ferromagnetic properties with suitable shape anisotropy. In these experiments, the nominal turbopump speed is 260 L/s for nitrogen gas. When the leak valve is opened, the chamber pressure increases. The chamber pressure is monitored through a Penning vacuum gauge, which serves to perform systematic quantitative studies (we cannot provide direct values of precursor flux on the substrate because the pumping efficiency of the vacuum pump for Fe_2_(CO)_9_ molecules in our apparatus is unknown). The beam current and voltage used for the growth were, respectively, 1.4 nA and 3 kV. The first experiments under relatively high precursor flux (process pressure approx. 6 × 10^−6^ mbar) indicated that the microstructure of the deposits consisted of grains with a typical size of about 100 nm, as can be observed in Figure S1 of Supporting Information File 1. In those wires, there was no significant modification of the coercive field as a function of the deposit dimensions, indicating that the magnetization reversal was governed by the individual grains, somehow magnetically decoupled one from each other. However, when the process pressure was decreased to the range from 3 × 10^−6^ to 4 × 10^−6^ mbar, the deposits did not show the granular structure and the magnetization reversal was found to be dominated by shape anisotropy (see next section). In situ compositional analysis by energy dispersive X-ray spectroscopy (EDS) indicated that the Fe content in these optimized deposits were always in the range of 80–85%, the rest being C and O. These values are similar to those found by Gavagnin et al. with the precursor Fe(CO)_5_ [18]. In Figure S2 of Supporting Information File 1, we show one of the typical EDS spectra of these deposits.

In the following, we will describe the results obtained in two batches of samples corresponding to optimized nanowires. In both batches, the length is fixed to 4.5 μm and the ends are triangular (see Figure 1c), as frequently used in this type of nanowires to avoid easy nucleation of domain walls at the end of the nanowire and thus the appearance of low coercive fields. In the first batch, *t*_Nom_ is fixed to 25 nm and the width is varied from 50 to 500 nm (4 samples). In the second batch, the width is fixed to 250 nm and *t*_Nom_ is varied from 10 to 45 nm (8 samples). This will allow for a systematic study of the effect of varying width and thickness in these nanowires.

### MOKE experiments: coercive field

Magneto-optical Kerr effect (MOKE) experiments have been carried out using the Nano-MOKE-3 apparatus by Durham Magnetooptics. This device uses a 2 μm diameter infrared laser beam that scans the area of interest in raster mode to detect MOKE contrasts via the variation of the signal amplitude or the change in the rotation angle of the light. The positioning of the beam permits the localization of the nanostructure to be measured and to position the beam in a targeted place. For measurements as a function of the magnetic field, the laser beam is fixed on the centre of the nanowire and quadrupole coils are used to apply a magnetic field in the plane of the sample, which allows for tracing the MOKE changes versus the magnetic field. The MOKE signal is proportional to the total magnetization, allowing one to study magnetization reversal processes and precise determination of the coercive field of the nanowires. In our experiments, we have measured the longitudinal MOKE signal with the magnetic field being applied parallel to the nanowire length (easy axis) [32].

**Figure 2:**
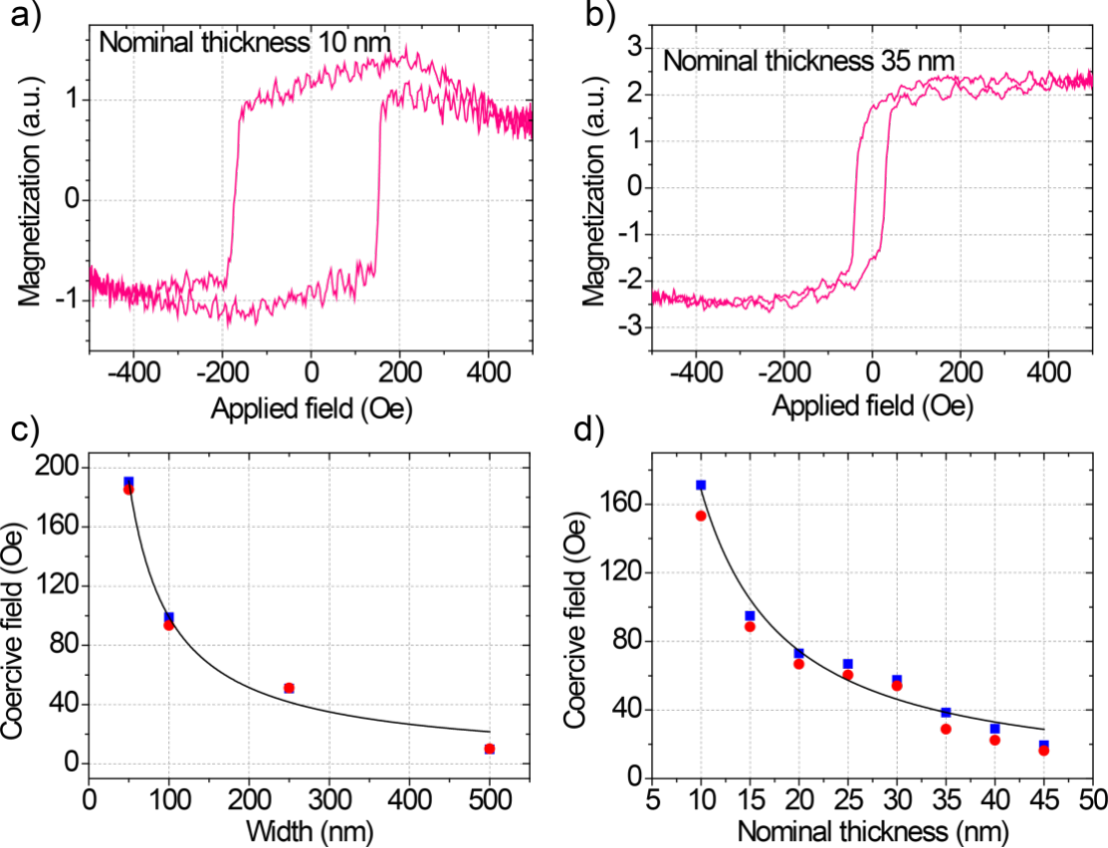
MOKE results. (a) Average magnetic hysteresis loop of the sample with width/nominal thickness of 250 nm/10 nm. (b) Average magnetic hysteresis loop of the sample with width/nominal thickness of 250 nm/35 nm. (c) Coercive field as a function of width for batch 1 (*t*_Nom_ = 25 nm) with positive and negative applied magnetic field (blue squares and red circles respectively). Please, be aware that the red circles partially hide the blue squares. The line is a fit to Equation 1. (d) Coercive field as a function of *t*_Nom_ for batch 2 (width = 250 nm) with positive and negative applied magnetic field (blue squares and red circles respectively). The line is a visual guide.

As an example of the type of magnetization loops measured, we show in Figure 2a and Figure 2b the results for two nanowires from the second batch with *t*_Nom_ of 10 and 35 nm. The signal-to-noise ratio is ten, certainly enough to determine the coercive field, which is given by the measured MOKE field at the mid-point between the two saturation values at high positive and high negative fields. The observed noise, about 10% of the signal, is small given that the nanowire width is only 1/8 of the laser diameter (thus having significant signal originated from the non-magnetic substrate). The values of *H*_C_ for both batches are represented in Figure 2c and Figure 2d. All results shown in Figure 2 correspond to the average of more than 100 hysteresis loops. Given the sharp switching transitions observed, a high level of reproducibility can be inferred.

The observed decrease of *H*_C_ with the width was also observed in polycrystalline cobalt nanowires grown by FEBID with widths above 250 nm [26–27] and previously in permalloy [33] and cobalt [34–36] nanowires patterned by electron beam lithography. Such dependence was explained by a model in which a small volume in the wire reverses magnetization coherently, propagating across the entire wire. In such a model, the coercive field is proportional to the ratio thickness (*t*)*/*width (*w*) due to demagnetizing effects:

[1]
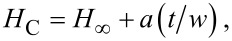


where *H*_∞_ is the coercive field for a wire with infinite width (thin film) and *a* is constant parameter that depends of the finite-length shape anisotropy factor and saturation magnetization [37]. Applying this model to the Fe wires of the batch 1, we obtain that *H*_∞_ is 7 Oe, indicating that the material is soft magnetic, as expected for Fe.

We note, however, that the model is not able to explain the thickness dependence of *H_C_* in the 250 nm wide Fe nanowires of the batch 2. In fact, we observe that *H*_C_ decreases with increasing thickness, contrary to the model. Similar behavior has also been observed theoretically in permalloy nanowires [38] and experimentally in permalloy nanowires arrays [39] and in cobalt nanowires grown by FEBID [40]. In all cases, the occurrence of the failure of the model is caused by a transition in the type of magnetization reversal process, which led us to perform micromagnetic simulations to investigate this possibility. Given the previous knowledge regarding the influence of the halo [31] and the effective magnetic shape [27] in the coercive field of FEBID magnetic nanostructures, it is convenient to know these properties in the studied nanowires. This is why we first carried out transmission electron microscopy (TEM) experiments to investigate the exact shape and composition of the nanowires, so that the subsequent micromagnetic calculations could reliably reproduce the observed dependence of the coercive field.

### TEM experiments: deposit shape, halo extension, surface oxidation

TEM characterization of the microstructure and composition of Fe nanowires was carried out in a probe-corrected FEI Titan 60-300 operated at 300 kV. Two TEM lamellae of Fe nanowires with a width of 250 nm and nominal thicknesses of 10 and 45 nm were fabricated using a focused Ga^+^ ion beam and standard lift-out procedures in an FEI Helios 600 Nanolab. The slices were cut perpendicular to the nanowire length to analyze their cross-sectional width profile. The morphology and microstructure were determined by bright field (BF) TEM and high resolution TEM (HRTEM) imaging, and chemical composition of the sections was determined by combining high angle annular dark field (HAADF) imaging and electron energy loss spectroscopy (EELS) in scanning transmission electron microscopy (STEM) mode. The beam current in the STEM-EELS experiments was 250 pA. Focused beam induced annealing effects were not observed in the experiments. These are easily observable because the images change with time, which was not the case in the experiments presented here.

**Figure 3:**
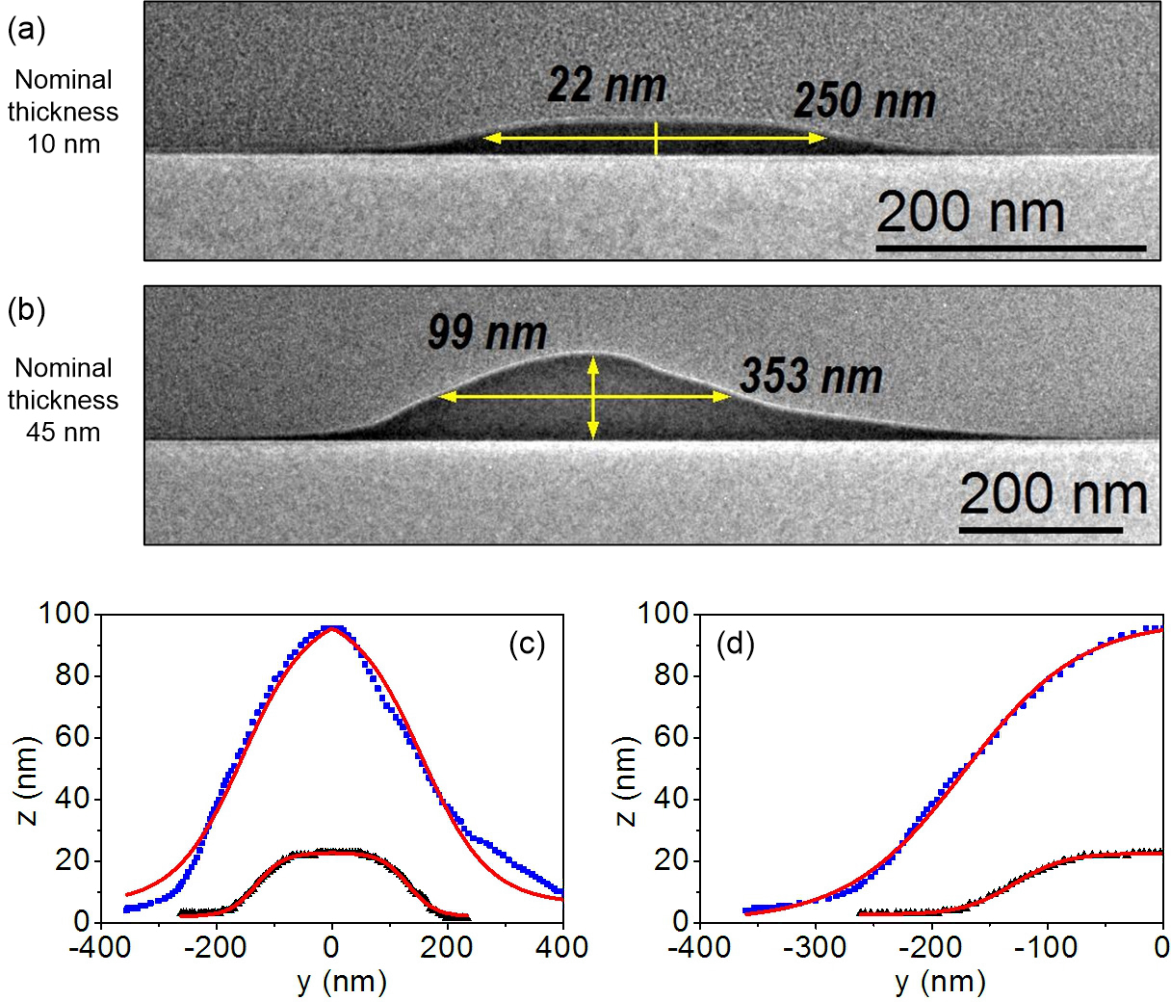
Low-magnification TEM images of the iron nanowires with width of 250 nm and nominal thickness of (a) 10 and (b) 45 nm. Fitting of the (c) full and (d) half profiles of both nanowires using Equation 2.

Low-magnification BF-TEM images of the cross-sections of Fe nanowires are shown in Figure 3a and Figure 3b. These wires have been chosen for the TEM study because they are respectively the thinnest and the thickest ones for the fixed width of 250 nm. Both wires presented irregular bell-shaped profiles, which are fitted using the following empirical formula:

[2]
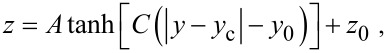


where *A* and *C* are fitting parameters while *y*_c_ corresponds to the peak position in the lateral *y* direction (in this case manually centered, *y*_c_ = 0) and 2*y*_0_ corresponds to the full width of the profile measured along the *y* direction at a height of *z*_0_. Mathematically speaking, *z*_0_ will correspond to the height where the bell profile changes its concavity (inflexion point). As one can notice in Figure 3c and Figure 3d, Equation 2 fits well the profiles of the nanowires extracted from TEM images of Figure 3a and Figure 3b. A better fit is obtained if we only take into account a half part of the profiles. All fitted parameters of both cases are reported in Table S3 (Supporting Information File 2). Comparing the values obtained from the fit for 2*y*_0_ and *z*_0_, one can note that both correlate, respectively, with the nominal values of the width and thickness of these two nanowires, being more similar for the thinnest one. In addition, the TEM images clearly reveal that the bell-shaped profile of the nanowires makes that the *t*_Nom_ is almost half of the maximum thickness (*t*_Max_), corresponding to the peak height. Taking this fact into account and given that we have experimentally measured the profiles of the thinnest and the thickest nanowires, a linear extrapolation of the fitting parameters of Equation 2 permits to estimate the profile parameters of the intermediate thicknesses. The values of this extrapolation are reported in Table S2 (Supporting Information File 2), considering the fitted parameters obtained from Figure 3d (half profile) and establishing *z*_0_ as the *t*_Nom_ of the nanowires. The HRTEM images of the nanowires, shown in Figure S3 (Supporting Information File 1), indicate that the Fe is nanocrystalline, as previously reported [14,18]. This microstructure will produce negligible magnetocrystalline anisotropy effects and, as a consequence, shape anisotropy will determine the magnetic anisotropy of the wires. The Fe content determined by EELS inside the wires is around 85%, in good agreement with the EDS performed inside the FIB-SEM equipment. According to previous studies, the saturation magnetization in Fe deposits grown by FEBID corresponds well with the Fe content [14,41]. Then, one would expect a saturation magnetization about 80–85% of that of bulk Fe.

**Figure 4:**
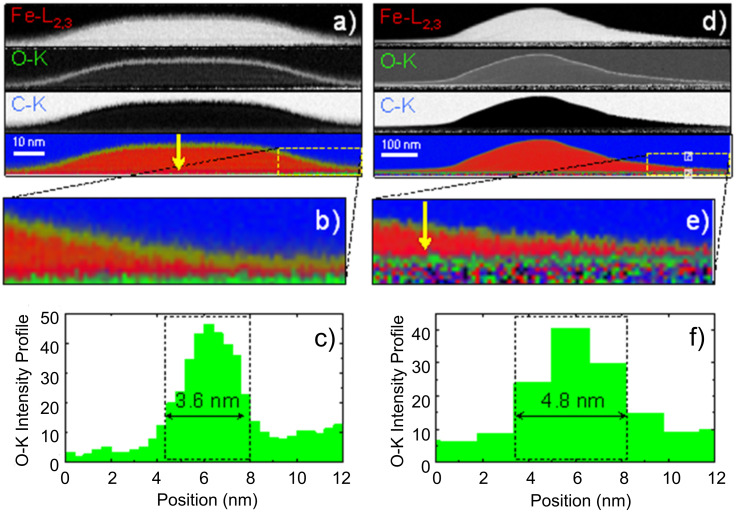
Compositional analysis through EELS of the iron nanowires with nominal thickness of (a) 10 nm and (d) 45 nm. In (b) and (e) a close-up of the corresponding halos is shown. In (c) and (f) the intensity profiles of the oxygen from above the surface towards the interior part of the two nanowires (at the positions marked with yellow arrows) are shown: The thickness of the oxidized surface layer (FeO) is around 4–5 nm.

From the STEM-EELS experiments shown in Figure 4, where the oxygen intensity is probed from above the surface towards the interior part of the nanowires, it can be concluded that the top surface of the nanowires exhibits an oxidized layer of 4–5 nm thickness. The stoichiometry of this oxidized layer is found to be Fe/O = 1:1 (49.4 ± 2% of Fe and 50.6 ± 2% of O), which corresponds to a paramagnetic material at room temperature. Such an oxidized surface is not ferromagnetic, which affects the overall magnetization reversal of the wires. The oxidized layer will have a strong impact on the thinner part of the wires, i.e., the tails and halo, which will be prone to lose the ferromagnetism. As a consequence, the effective ferromagnetic volume of the wire will be more localized towards the centre of the nanowire, thus modifying its functional ferromagnetic shape.

### Micromagnetic simulations

Quasi-static micromagnetic simulations were carried out to study the influence of the shape profile of the Fe nanowires and oxidized surface layer on the magnetization reversal process. We have focused our attention on the dependence of *H*_C_ on the thickness, which deviates from the model of magnetization reversal for nanowires described by Equation 1. In particular, we have studied the variation of *H*_C_ with the thickness for 250 nm wide Fe nanowires considering the actual geometry of the Fe nanowires of batch 2. The GPMagnet software package [42] was used to perform the simulations employing the following magnetic parameters for polycrystalline pure iron [43]: saturation magnetization = 1.7 × 10^6^ A/m, exchange constant = 2.1 × 10^−11^ J/m, anisotropy constant = 0 (we assume that in nanocrystalline iron the magnetocrystalline anisotropy is averaged out). For the simulation, we have adopted the same in-plane geometry used for the MOKE measurements shown before. In order to decrease the simulation time, different cell sizes have been used depending on the thickness (see Table S1 in Supporting Information File 2). *H*_C_ has been calculated for three different cases, which allows us to investigate in a more general way the influence of the shape of the nanowires in the behavior of the coercive field:

**case I:** a rectangular profile where constant width (250 nm) is considered, **case II:** the actual bell-shaped profile determined from the TEM measurements, and **case III:** a reduced bell-shaped profile considering a surface oxidation layer of 5 nm along the whole deposit.

**Figure 5:**
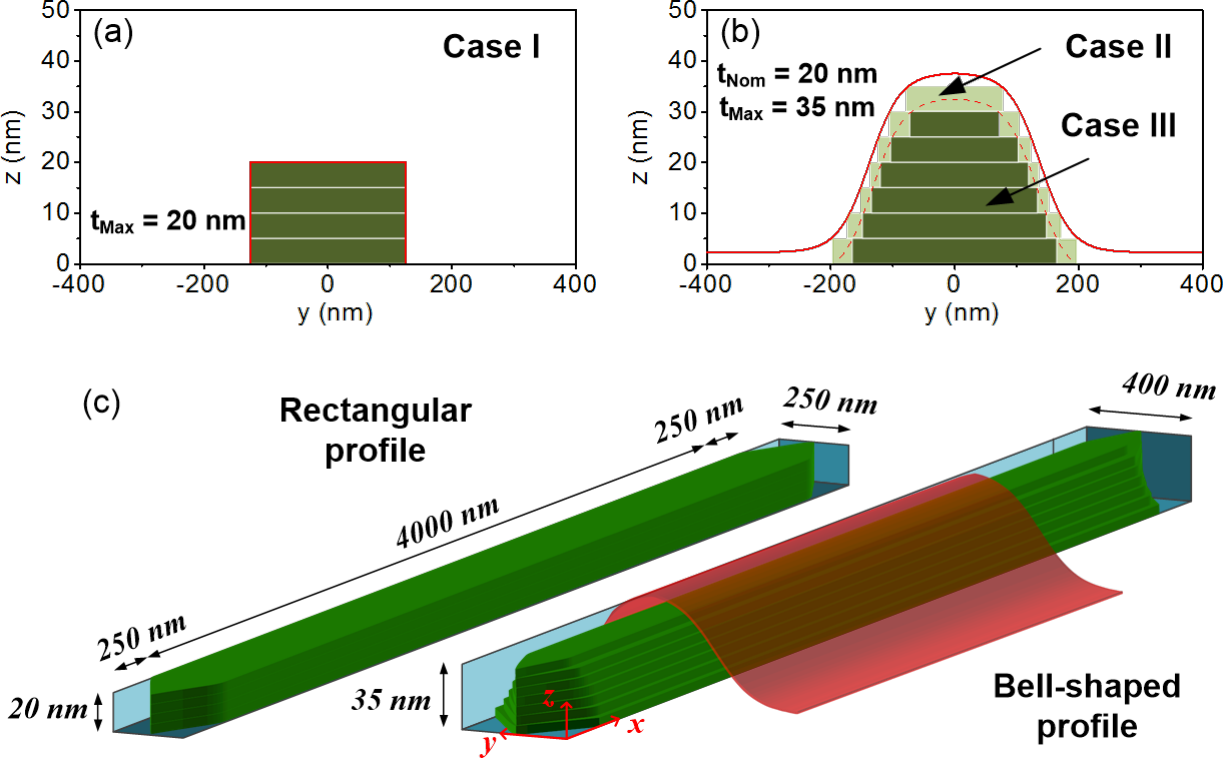
Sketch of the two-dimensional (*y*,*z* plane) geometrical shapes used in the micromagnetic simulation for a 250 nm wide Fe nanowire with a *t*_Nom_ of 20 nm: (a) Rectangular profile (case I), (b) approximated bell-shaped profiles without (case II) and with (case III) an oxidized surface layer of 5 nm. (c) Three-dimensional representation of the same wire in case I (left) and case II and case III (right).

The three different cases are schematically shown in Figure 5 for the Fe nanowire with a *t*_Nom_ of 20 nm. As one can notice in Figure 5, in case I a single value of thickness can be considered, corresponding to the maximum height of the rectangular profile (i.e., *t* = *t*_Nom_ = *t*_Max_). However, in the nanowires of cases II and III we defined *t*_Nom_ = *z*_0_ and *t*_Max_ = *z*_Max_ (peak height). The three-dimensional (3D) shape of the nanowires was designed by stacking several layers of equal thickness and either using the same area in the *x*,*y* plane (for case I) or progressively reducing their widths in order to approach the bell-shape of the profile (for case II and III). The sketch shown in Figure 5c provides a visual understanding of the 3D structure of the simulated Fe nanowires. To simulate the oxidized bell-shaped profile, the structural bell-shaped profile has been reduced 5 nm from the surface in order to keep only the magnetic volume. It should be pointed out that temperature is not taken into account in the simulations. This is why the absolute value of *H*_C_ in the simulations is expected to be twice or more the experimental value at room temperature [27].

**Figure 6:**
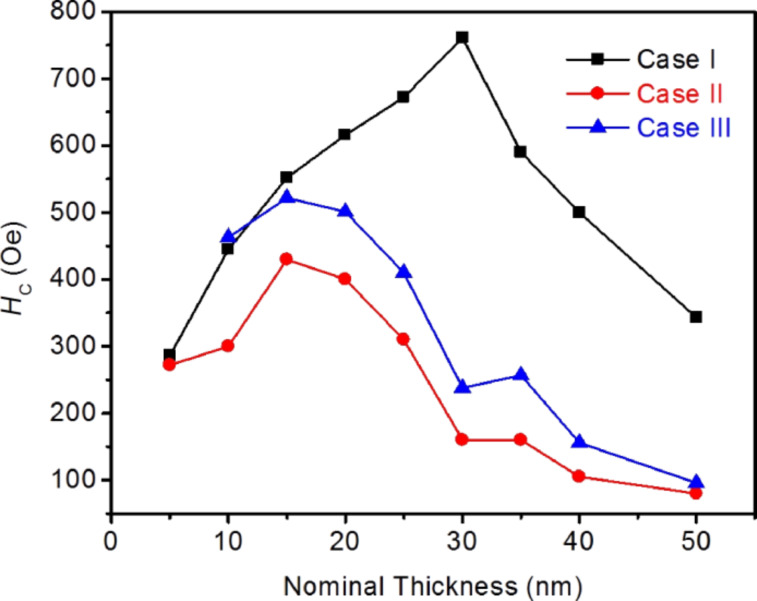
Coercive field (*H*_C_) obtained from the simulations of iron nanowires with nominal width of 250 nm and varying nominal thickness (from 5 to 50 nm) for the three cases discussed in the text.

Plots of the simulated *H*_C_ values as a function of the nominal thickness for the three cases considered are presented in Figure 6. For case I (rectangular profile), in the range of thickness 5 nm < *t*_Nom_ < 30 nm, one notices that *H*_C_ increases with the thickness, as expected from Equation 1, until reaching a critical thickness (*t*_c_) of 30 nm. At that point, *H*_C_ exhibits a maximum. Above *t*_c_, *H*_C_ decreases with thickness. A similar behavior is observed in the nanowires with bell-shaped profiles, with and without the oxidized top layer of 5 nm (cases II and III, respectively). However, one can notice that the bell-shaped profile favors the occurrence of the maximum value of *H*_C_ at a lower *t*_Nom_ (*t*_c_ = 15 nm, for both cases II and III), also reducing the values of *H*_C_ for the same *t*_Nom_ compared to case I. Above *t*_C_, we note that the decreasing of *H*_C_ with the thickness observed in the nanowires of cases II and III resembles the experimental result (shown in Figure 2d). The full behavior of the simulated *H*_C_ as a function of the thickness in the bell-shaped Fe nanowires resembles the one that is reported for 500 nm wide L-shaped Co-FEBID nanowires [40]. In [40], a maximum value of *H*_C_ is obtained at the crossover between two types of DWs nucleated in the corner of the “L”. The transition is from transversal DWs (at low thicknesses) to vortex DWs (at high thicknesses). In principle, a change in the magnetization reversal mode is a good candidate to produce a dependence of *H*_C_ with thickness presenting a maximum. The micromagnetic simulations in the Fe nanowires permit a direct visualization of the magnetic configuration during magnetization reversal, which allows us to explore the changes in the reversal magnetization mechanism between nanowires with low and high thickness.

**Figure 7:**
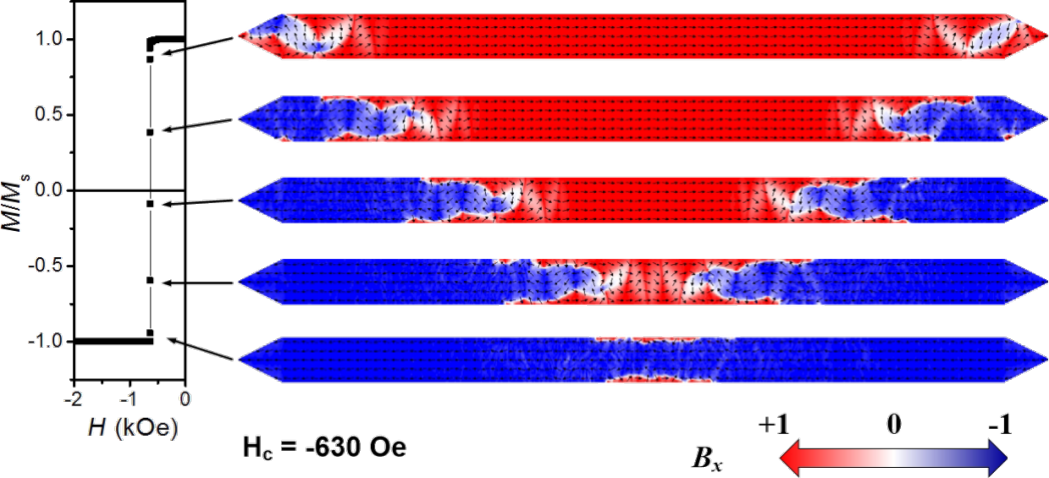
Simulated magnetization reversal with five snapshots of the magnetization state in the nanowire with width of 250 nm, thickness of 20 nm and profile defined as case I.

In Fe nanowires with rectangular profile (case I), for values of thickness *t*_Nom_ < *t*_c_ (*t*_c_ = 30 nm), we find that the reversal magnetization process occurs through the propagation of two extended domain walls (EDWs). These relatively complex head-to-head and tail-to-tail EDWs are first nucleated at the ends of the nanowire and then propagate along the nanowire until they meet at the nanowire center and annihilate. Figure 7 shows five snapshots of the magnetization reversal process in the sample with width of 250 nm, thickness of 20 nm and profile defined as case I. This magnetization reversal mechanism is not via the formation of a multi-domain structure but monodomain-type, with EDW nucleation at the pointed ends of the nanowires and propagation towards the center.

**Figure 8:**
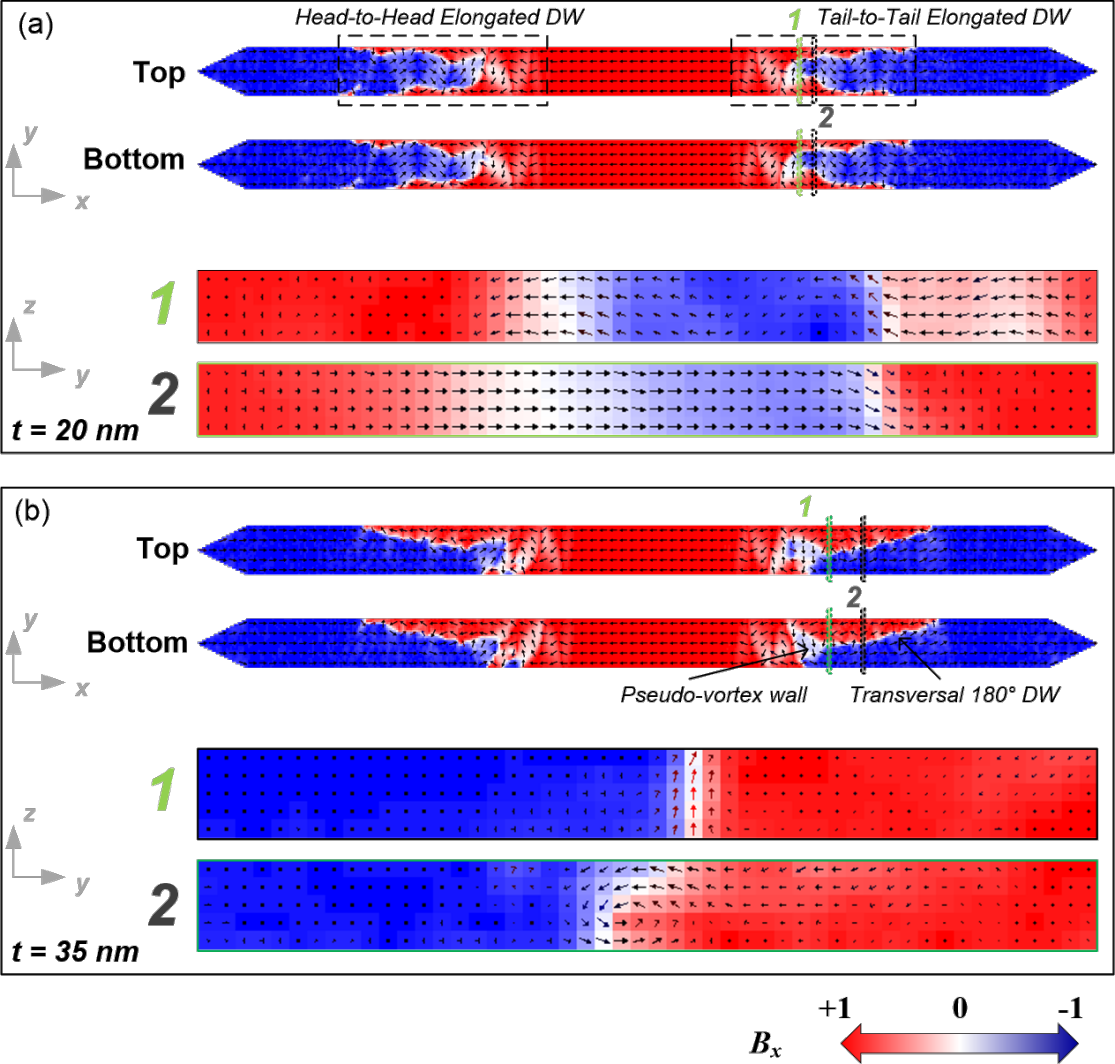
Magnetization vector-color maps extracted from the simulations for 250 nm wide Fe nanowires with rectangular profile (case I) and thickness of (a) 20 nm and (b) 35 nm. The areas named “1” (green lines) and “2” (black lines) in the *x*,*y* plane representations have been chosen for the representation in the *y*,*z* plane.

Interestingly, the simulations performed give access to image the *x*,*y* plane-view magnetization states along the full depth of the nanowires. One example is the top and bottom layers of the 20 nm thick nanowire with rectangular profile, shown in Figure 8a. The EDWs observed in this nanowire consist of small (extending only locally) 180° and 90° DWs, where the local magnetization rotates coherently in the plane of the nanowire (*x*,*y* plane) without out-of-plane magnetization rotations. This is in agreement with the observed magnetization reversal through coherent rotation modes that occurs at low thickness of magnetic nanowires [39,44]. This behavior can also be observed in cross-sectional images of the magnetization extracted along an EDW (*y*,*z* plane), where the local magnetization rotation across the whole thickness of the DWs occurs in the *x,y* plane (see the *y*,*z* plane view included in Figure 8a). Thus, the magnetic structure of the EDW is composed by small DWs covering the whole thickness and propagating to produce the magnetization reversal. This magnetization reversal mechanism, corresponding to a coherent rotation mode*,* is theoretically well described by Equation 1 [37]. Therefore, an increasing value of *H*_C_ with the thickness is expected in that nanowire thickness range. For *t*_c_ < *t*_Nom_ < 50 nm simulations also indicate a monodomain-type reversal magnetization, through nucleation and propagation of EDWs, even though their small constituent DWs present local non-coherent rotation modes. This effect can be noticed in the cross-sectional images of the magnetization extracted across the EDWs for the 35 nm thick Fe nanowire of rectangular profile depicted in Figure 8b. Both, the pseudo-vortex wall formed in the inner part of EDW structure and the extended transversal 180° DW formed in the external part of the EDW structure, reveal local out-of-plane magnetization rotations of either Neél-type or C-shape magnetization distributions. In thicker films (above *t*_c_) the formation of DWs with non-coherent magnetization rotation is energetically more favorable than having coherent magnetization rotation [44–45]. The crossover to non-coherent magnetization reversal modes will be accompanied by a decrease of the DWs energy with the thickness. A direct consequence of this fact is that *H*_C_ will decrease with the thickness, as experimentally observed in Figure 2d.

**Figure 9:**
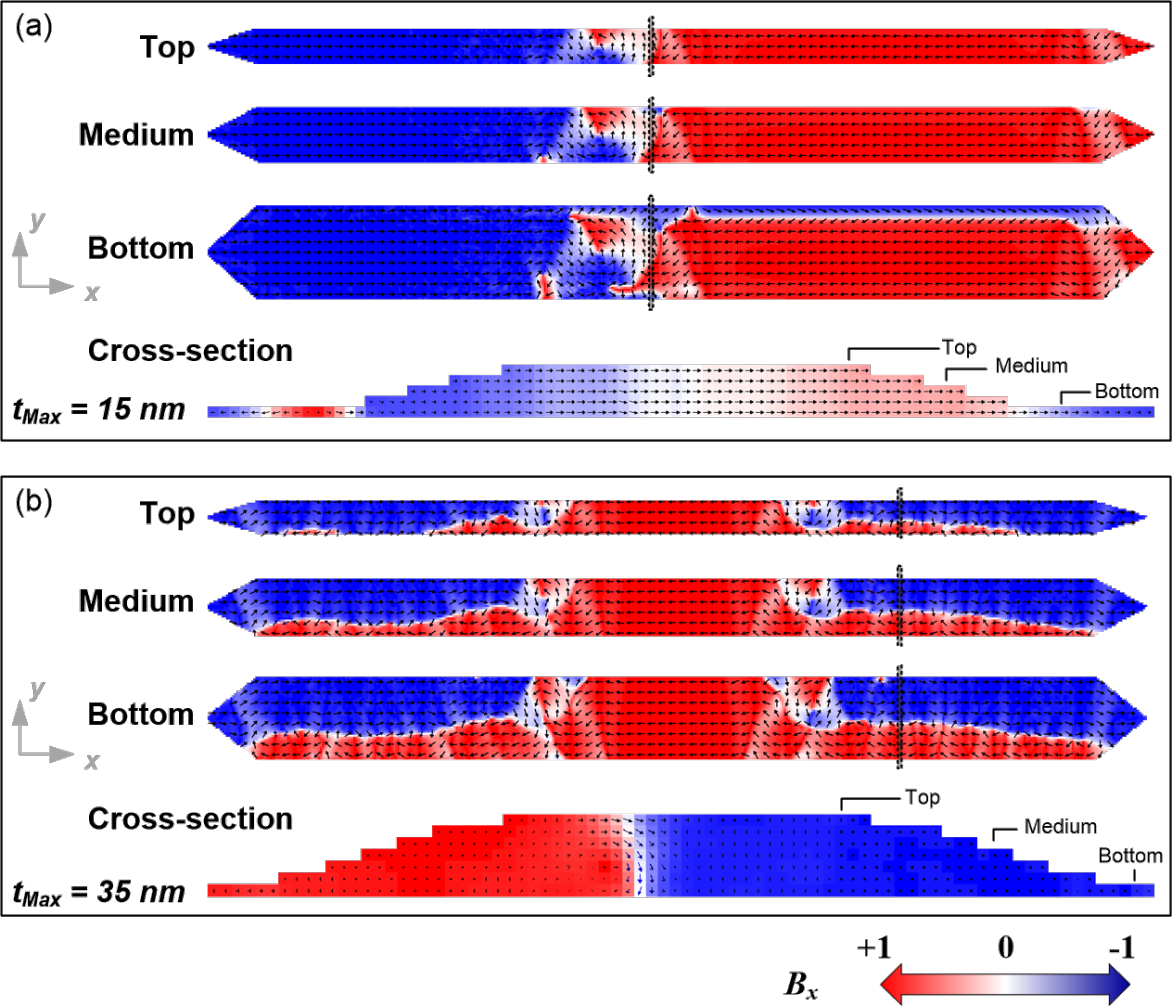
Magnetization vector-color maps extracted from the simulations for 250 nm wide Fe nanowires with bell-shaped profile (case II) and nominal thickness of (a) 10 nm and (b) 20 nm. The areas marked with black lines in the *x*,*y* plane representations have been chosen for the representation in the *y*,*z* plane (cross-section).

The same reversal magnetization mechanisms previously observed in the rectangular profile nanowires have been found in the bell-shaped profile nanowires (case II and III). Thus, for *t*_Nom_ < *t*_c_, with *t*_c_ = 15 nm, a monodomain-type reversal magnetization of EDWs with coherent rotation occurs, as shown in Figure 9a for a bell-shaped profile nanowire (case II) with *t*_Nom_ = 10 nm. For *t*_c_ < *t*_Nom_ ≤ 25 nm, a monodomain-type reversal magnetization of EDWs with non-coherent rotation occurs, as shown in Figure 9b for a bell-shaped profile nanowire (case II) with *t*_Nom_ = 20 nm.

**Figure 10:**
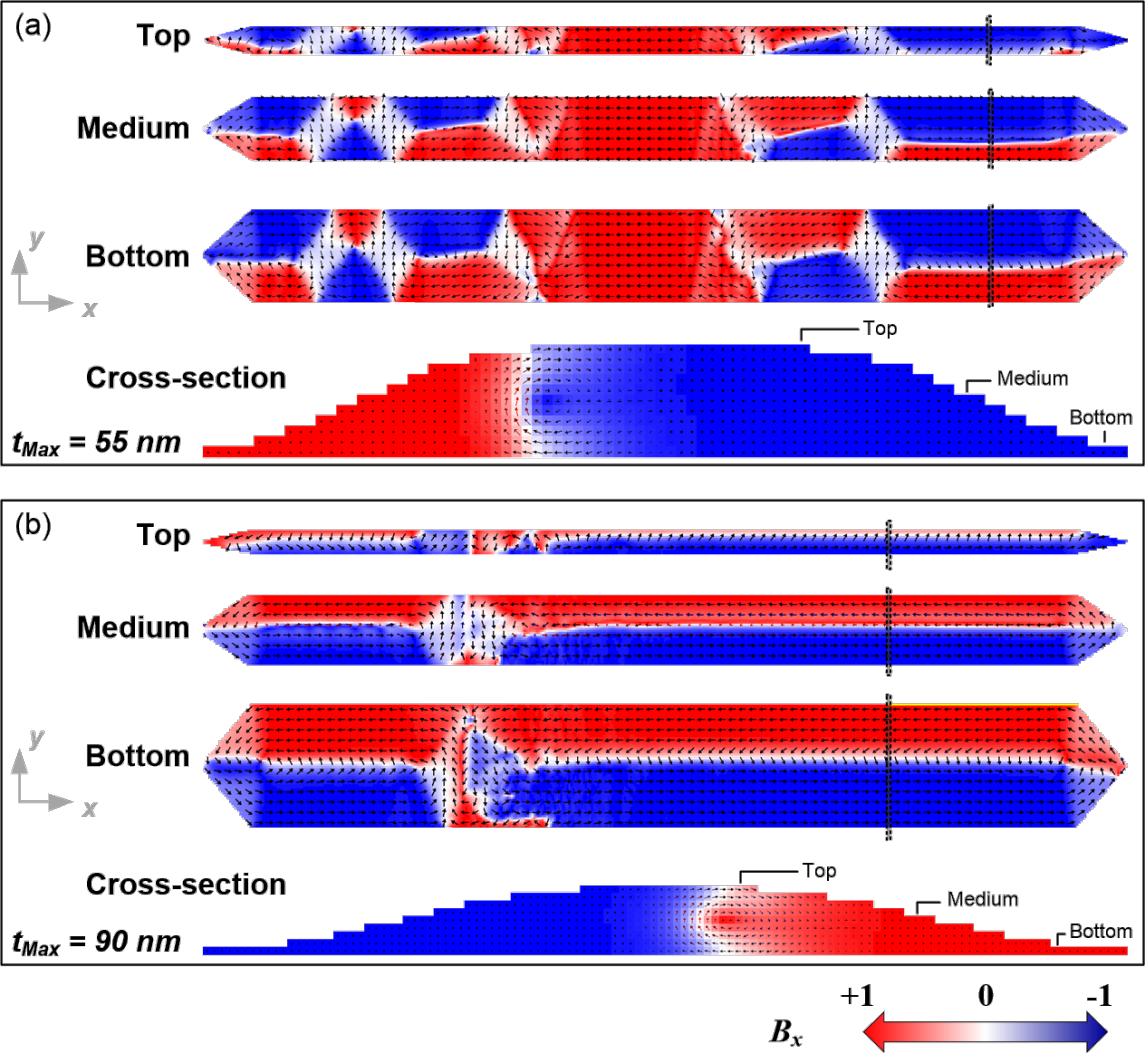
Magnetization vector-color maps extracted from the simulations for 250 nm wide Fe nanowires with bell-shaped profile (case II) and nominal thicknesses of (a) 30 nm and (b) 50 nm. The areas marked with black lines in the *x*,*y* plane representations have been chosen for the representation in the *y*,*z* plane (cross-section).

Interestingly, for 30 nm ≤ *t*_Nom_ ≤ 50 nm, the reversal magnetization process is already multidomain-type, as shown in Figure 10. Indeed, for nominal thicknesses of 30 and 35 nm, the switching is produced through the formation and displacement of several magnetic domains along the nanowire length. The example of the 30 nm nanowire is shown in Figure 10a. For *t*_Nom_ = 40 and 50 nm, the magnetization reversal is given by the displacement of two transversal 180° DWs, each of them initiated at one of the pointed ends of the nanowires. In this displacement, while one extreme of the transversal 180° DW is pinned at the apex of the nanowire, the other extreme moves towards the center of the nanowire. When both DW meet at the center, they form a single DW spanning along the full length of the nanowire, forming a Landau–Liftshitz domain pattern, as displayed in Figure 10b.

**Figure 11:**
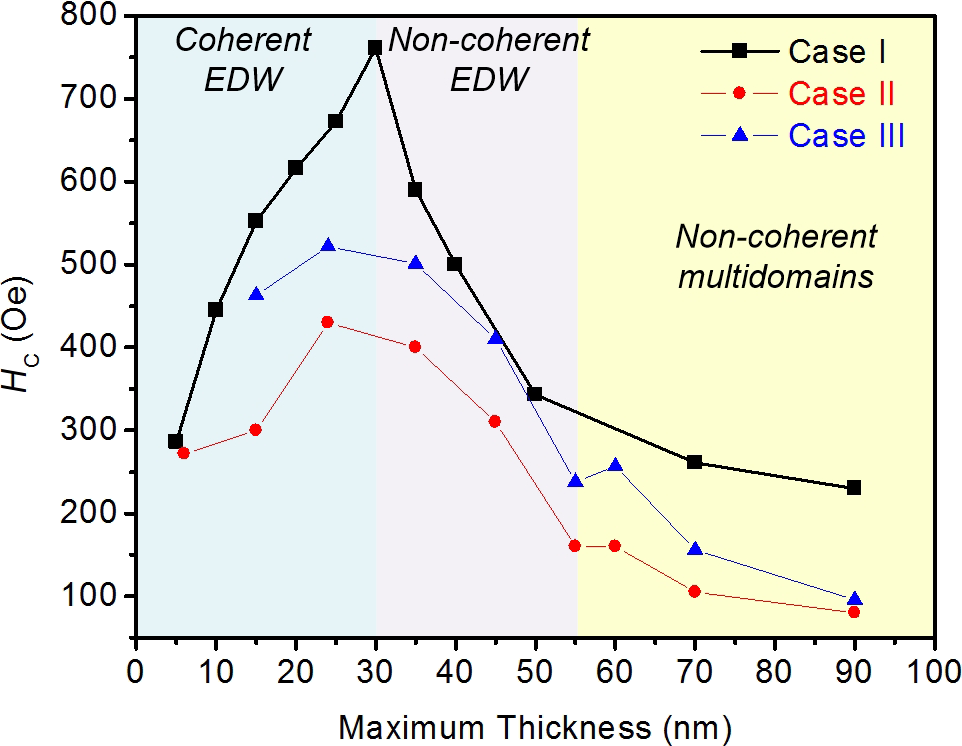
Coercive field (*H*_C_) obtained from the simulations of nanowires as a function of *t*_Max_ for the three cases.

An interesting behavior is observed if we plot the values of *H*_C_ as a function of *t*_Max_. This new representation, shown in Figure 11, indicates that the different reversal magnetization mechanisms are directly linked to the maximum thickness, independently of the shape of the profiles. The shape of the profile, however, allows for the tuning of the value of *H*_C_, being higher in the rectangular profile nanowires. The surface oxidation in the bell-shaped profile (giving rise to a reduction of the magnetic volume) produces an increase in the values of *H*_C_. In Figure 11, two additional values of *H*_C_ for rectangular profile nanowires with *t*_Max_ = *t*_Nom_ = 70 and 90 nm are reported. The simulations show a non-coherent multidomain magnetization reversal in such nanowires, similar to that observed in the bell-shaped profile nanowires in the same *t*_Max_ range.

From the information obtained in the micromagnetic simulations, one can safely infer that the dependence of *H*_C_ on the thickness in Fe nanowires is directly related to the specific magnetization reversal mode taking place at that thickness range. While reversal modes via coherent magnetization rotation produce an increasing value of *H*_C_ with the thickness, reversal modes via non-coherent magnetization rotation make *H*_C_ decrease with the thickness. Extrapolating this analysis to the experimental results obtained, we conclude that the decreasing value of *H*_C_ with thickness observed in Figure 2d is caused by magnetization reversal modes with non-coherent magnetization rotation.

Having established the reason for the dependence of *H*_C_ on the thickness over the thickness range studied, a number of interesting features need to be discussed.

**i) Effects produced by the bell shape and the halo of the nanowires.** As shown in Figure 6, the bell-shaped nanowires present a lower *H*_C_ compared to the ones with a rectangular profile. This was already found in the simulations of FEBID Co nanowires performed by Fernández-Pacheco et al. [27]. The reason is that the nucleation of DWs is favored at the thinnest parts of the nanowire, which facilitates the magnetization reversal. Previous simulations in Fe nanowires considering an extended halo also highlighted its influence on the magnetization reversal [31]. Moreover, experiments where the halo was eliminated by means of ion irradiation already indicated its relevant role played in the magnetization reversal [28–29].

**ii) Effects produced by the oxidized top layer of 5 nm.** The oxidized top layer of 5 nm is expected to play some role in thin nanowires due to the modification of the magnetic properties of a substantial part of the nanowire. Assuming that this oxidized layer is paramagnetic at room temperature (as the Fe/O 1:1 stoichiometry suggests), this surface layer will lose its ferromagnetic behavior. According to the simulations shown in Figure 6, the main effect of such oxidized layer is therefore to increase *H_C_*. This is explained by the change in the ferromagnetic shape of the nanowire caused by the oxidation, which degrades or annihilates the ferromagnetism in the halo. The halo is a source for easy DW nucleation and its oxidation will weaken such mechanism for the initiation of the magnetization reversal.

**iii) Behavior of *H*_C_ at low thickness.** The simulations predict that for the thinnest bell-shaped nanowires (*t*_Nom_ < 15 nm), a regime of increasing *H*_C_ with thickness could be observed. This thickness regime was not experimentally addressed by us in a systematic way as it was beyond the scope of the present work. In fact, only one sample in our study falls in that thickness range, which does not permit to extract any reliable conclusion. However, it seems an interesting focus for future work. Such challenging work should consider, for example, a systematic characterization of the iron oxides formed at the surface, the continuity of the magnetic layer at such low thicknesses, and the nanowire roughness.

## Conclusion

A systematic magneto-optical Kerr effect study of the coercive field as a function of thickness and width in Fe nanowires grown by focused electron beam induced deposition (FEBID) has been carried out. It has been found that the coercive field decreases for increasing thickness and width in the range of dimensions studied. In the particular case of *H*_C_ vs thickness for nanowires with constant width (250 nm), micromagnetic simulations have demonstrated that the decrease of *H*_C_ with thickness is due to the prevalent magnetization reversal mechanism, namely non-coherent magnetization reversal. In addition, micromagnetic simulations also show that the actual bell shape of the FEBID nanowires is important for the exact value of *H*_C_. The formation of a 5 nm surface oxidation layer on top of the nanowires has been observed experimentally. Micromagnetic simulations show that such surface oxidation produces a slight increase in *H*_C_. The results shown in this work demonstrate that *H*_C_ can be tailored in Fe nanowires grown by FEBID with appropriate control over their dimensions and shape, which is a crucial step towards the fabrication of functional devices based on these deposits such as magnetic memories, logic circuits and magnetic sensors. The reported micromagnetic simulations provide a detailed understanding of the magnetization reversal mechanisms in these Fe nanowires.

## Supporting Information

File 1 Structural and compositional characterization of the iron nanowires.

File 2 Additional information about the profile shape of the nanowires and micromagnetic simulations.
