# Supplementary material for: Influence of the shape and surface oxidation in the magnetization reversal of thin iron nanowires grown by focused electron beam induced deposition
Source: Beilstein J Nanotechnol. 2015 Jun 15;6:1319–31. doi: 10.3762/bjnano.6.136 (PMC4505150; doi:10.3762/bjnano.6.136)
Supplement: File 1 — Structural and compositional characterization of the iron nanowires. [file Beilstein_J_Nanotechnol-06-1319-s001.pdf]

Supporting Information

for

Influence of the shape and surface oxidation in the magnetization reversal of thin iron nanowires grown by focused electron beam induced deposition

Luis A. Rodríguez[$1,2,3], Lorenz Deen[$1,4], Rosa Córdoba[1,4], César Magén[1,2,5], Etienne Snoeck[3], Bert Koopmans[4] and José M. De Teresa*[1,2,6]

Address: [1]Laboratorio de Microscopias Avanzadas (LMA) - Instituto de Nanociencia de Aragón (INA), Universidad de Zaragoza, 50018 Zaragoza, Spain; [2]Departamento de Física de la Materia Condensada, Universidad de Zaragoza, 50009 Zaragoza, Spain; [3]CEMES-CNRS 29, rue Jeanne Marvig, B.P. 94347, F-31055 Toulouse Cedex, France; [4]Department of Applied Physics, Eindhoven University of Technology, PO Box 513, 5600 MB Eindhoven, Netherlands; [5]Fundación ARAID, 50018 Zaragoza, Spain and [6]Instituto de Ciencia de Materiales de Aragón (ICMA), Universidad de Zaragoza-CSIC, 50009 Zaragoza, Spain

Email: José M. De Teresa* - deteresa@unizar.es

* Corresponding author

[$]These authors have equally contributed to the work.

Structural and compositional characterization of the iron nanowires

In Supporting Information File 1, additional microscopic characterization of the Fe deposits is provided. As can be noticed in Figure S1, SEM images indicate that the microstructure of deposits grown under high precursor flux is granular. This hampers the observation of magnetic anisotropy, as discussed in the main text.

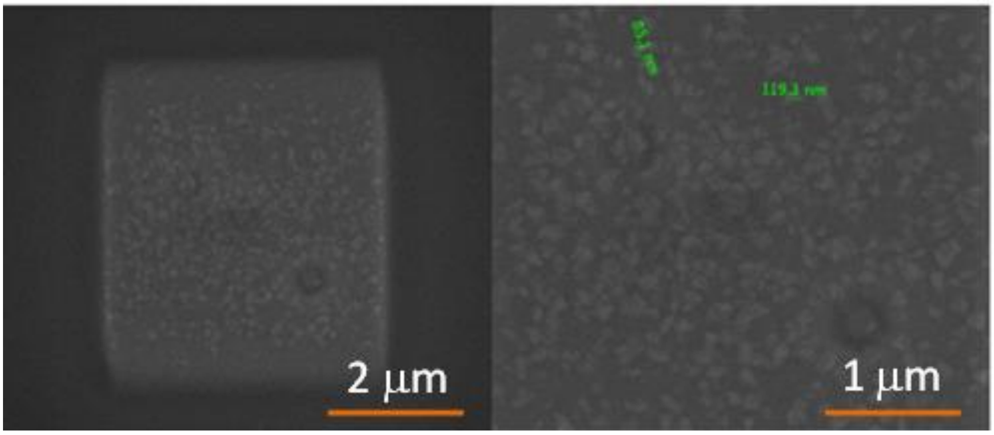

Figure S1: SEM image of the iron deposit grown at high precursor flux, process pressure of 6 × 10^{-6} mbar, showing the presence of grains with size larger than 100 nm.

One typical raw-data compositional analysis by EDS is shown in Figure S1_2. It corresponds to one of the deposits with optimized growth conditions. The quantitative analysis of the spectrum shows Fe content above 80%.

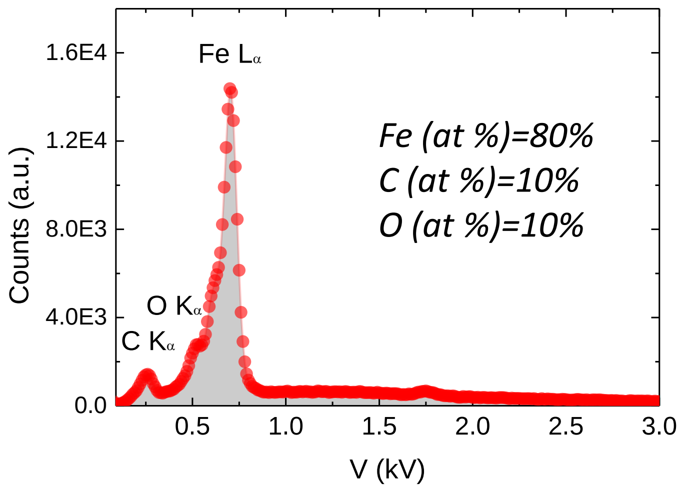

Figure S2: EDS spectrum of an optimized iron deposit.

In Table S1, characteristic dimensional values of the lateral dimensions of the thinnest and thickest Fe nanowires, obtained by means of TEM measurements, are shown.

Table S1: Characteristic values of dimensions of the nanowires.

	Width			Thickness			
**Nanowire**	**Nominal**	**FWHM**	**% difference**	**Nominal**	**Half Maximun**	**% difference**	**Maximun**
Thinnest	250 nm	250 nm	0%	10 nm	11 nm	10%	22 nm
Thickest	250 nm	353 nm	41%	45 nm	50 nm	11%	99 nm

The high-resolution TEM images of the thinnest and thickest Fe nanowires are shown in Figure S3. Their microstructure is mostly composed of an amorphous matrix, as deduced from the predominantly diffuse scattering in the Fast Fourier Transform (FFT). This amorphous matrix contains nanocrystals of approx. 1 to 2 nm size whose FFT corresponds with α-Fe.

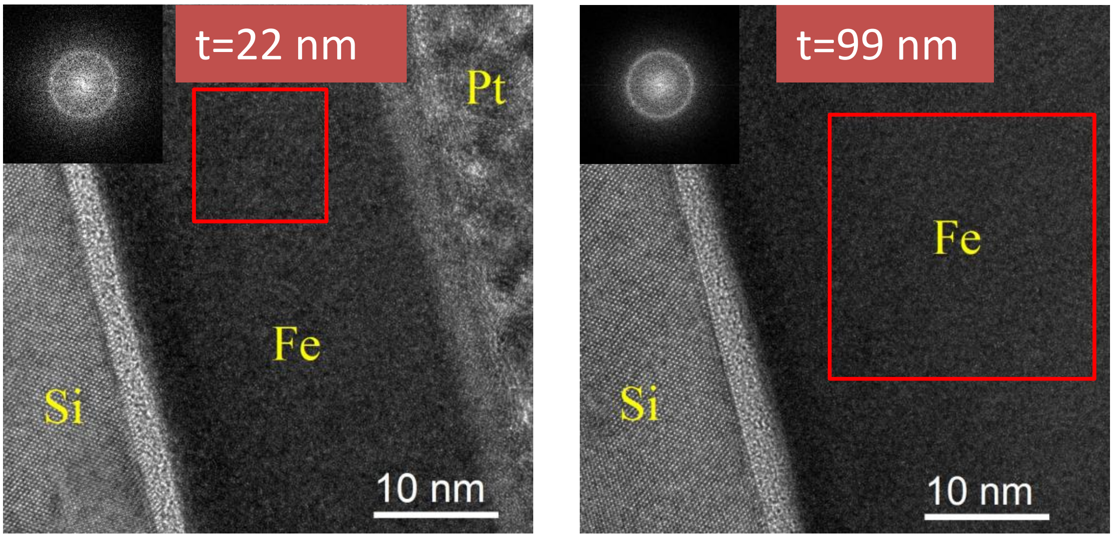

Figure S3: HRTEM images of two selected iron nanowires. The FFT of the squared red areas are shown at the top left of each image. The maximum thicknesses of each deposit (22 nm and 99 nm) are specified.

Examples of EELS spectra of the O K and Fe $L_{2,3}$ edges_used for compositional analysis are shown in Figure S4. This plot depicts a spectrum collected in the inner part of the thin (nominal 10 nm) iron nanowires, and one recorded in the outer surface, presenting a remarkable oxidation. They have been extracted from the spectrum images used for chemical mapping, shown in Figure 4 of the main manuscript text.

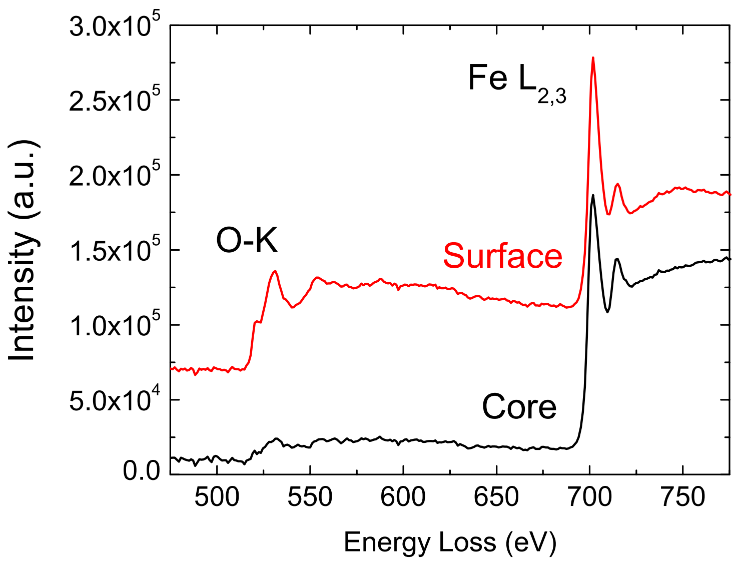

Figure S4: EELS spectra obtained at the core of the nanowire (black line) and at the nanowire surface (red line). They are plotted vertically shifted for the sake of clarity.
