# Supplementary material for: Influence of the shape and surface oxidation in the magnetization reversal of thin iron nanowires grown by focused electron beam induced deposition
Source: Beilstein J Nanotechnol. 2015 Jun 15;6:1319–31. doi: 10.3762/bjnano.6.136 (PMC4505150; doi:10.3762/bjnano.6.136)
Supplement: File 2 — Additional information about the profile shape of the nanowires and micromagnetic simulations. [file Beilstein_J_Nanotechnol-06-1319-s002.pdf]

# Supporting Information

for

# Influence of the shape and surface oxidation in the magnetization reversal of thin iron nanowires grown by focused electron beam induced deposition

Luis A. Rodríguez[$1,2,3], Lorenz Deen[$1,4], Rosa Córdoba[1,4], César Magén[1,2,5], Etienne Snoeck[3], Bert Koopmans[4] and José M. De Teresa*[1,2,6]

Address: [1]Laboratorio de Microscopias Avanzadas (LMA) - Instituto de Nanociencia de Aragón (INA), Universidad de Zaragoza, 50018 Zaragoza, Spain; [2]Departamento de Física de la Materia Condensada, Universidad de Zaragoza, 50009 Zaragoza, Spain; [3]CEMES-CNRS 29, rue Jeanne Marvig, B.P. 94347, F-31055 Toulouse Cedex, France; [4]Department of Applied Physics, Eindhoven University of Technology, PO Box 513, 5600 MB Eindhoven, Netherlands; [5]Fundación ARAID, 50018 Zaragoza, Spain and [6]Instituto de Ciencia de Materiales de Aragón (ICMA), Universidad de Zaragoza-CSIC, 50009 Zaragoza, Spain

Email: José M. De Teresa* - deteresa@unizar.es

* Corresponding author

[$]These authors have equally contributed to the work.

## Additional information about the profile shape of the nanowires and micromagnetic simulations

In Supporting Information File 2, additional information about the micromagnetic simulations is provided. In Table S1, we include the values of cell sizes used in the micromagnetic simulations using the GPMagnet package. The cell size is the size of the digitalization volume in the simulations. In other words, it is the voxel (volumetric pixel). Inside a cell, it is assumed that the magnetization is the same for all magnetic atoms. This digitalization has to be accommodated to the sample size in order to reproduce the real volume of the sample.

**Table S1:** Cell sizes used in the micromagnetic simulations.

| **CASE I (Rectangular profile)** | | **CASE II (Bell-shaped profile)** | | **CASE III (Bell-shaped profile + oxidized layer)** | |
|---|---|---|---|---|---|
| **Thickness (nm)** | **Cell Size ($nm^3$) x×y×z** | **Thickness (nm)** | **Cell Size ($nm^3$) x×y×z** | **Thickness (nm)** | **Cell Size ($nm^3$) *x* × *y* × *z*** |
| 5, 10, 15, 20, 25, 30 | 5 × 5 × 5 | 5, 10, 15, 20, 25, 30 | 5 × 5 × 5 | 5 | 5 × 5 × 2 |
| 35 | 5 × 5 × 7 | 35 | 5 × 5 × 7 | 10 | 5 × 5 × 3 |
| | | | | 15 | 5 × 5 × 4 |
| 40 | 5 × 5 × 20 | 40 | 5 × 5 × 20 | 20, 25, 30 | 5 × 5 × 5 |
| 50 | 5 × 5 × 25 | 50 | 5 × 5 × 25 | 35, 40, 50 | 5 × 5 × 10 |

As described in the main text, three different types of nanowire profiles were investigated (cases I, II and III). For the cases II and III, we used the parameters of the Table S2 to reproduce the profile shape for different thicknesses. Given that we only had experimental data of profiles of the samples with nominal thickness 10 nm and 45 nm (see Table S3), for the intermediate thicknesses we assumed a lineal dependence of each parameter with respect to thickness. In Figure S1, the generated profiles are represented.

For case II, $z_0$ and $2y_0$ have been respectively used to determine the thickness and the width for the rectangular profile taking the exact value of $z_0$ and the nearest multiple of 5 of $2y_0$, given the discrete size of the micromagnetic cells ($5 \times 5$ $nm^2$). For case III, we have simulated the bell-shaped profiles by stacking

rectangular layers where their thicknesses were related to the cell size and their widths were limited by the fitting function, $z(y)$, in Equation 2.

**Table S2:** Estimated fitting parameter for different thicknesses to reproduce cases II and III profile shapes.

| *Thickness* | A (nm) | $C$ (nm$^{-1}$) | $y_c$ (nm) | $2y_0$ (nm) | $z_0$ (nm) |
|---|---|---|---|---|---|
| ***5 nm*** | −1.84 | 0.0251 | 0 | 239.6 | 5 |
| ***10 nm*** | −7.84 | 0.0237 | 0 | 251.4 | 10 |
| ***15 nm*** | −12.41 | 0.0217 | 0 | 263.2 | 15 |
| ***20 nm*** | −17.70 | 0.0200 | 0 | 275.2 | 20 |
| ***25 nm*** | −22.98 | 0.0183 | 0 | 287.0 | 25 |
| ***30 nm*** | −28.27 | 0.0166 | 0 | 298.8 | 30 |
| ***35 nm*** | −33.55 | 0.0149 | 0 | 310.6 | 35 |
| ***40 nm*** | −38.84 | 0.0132 | 0 | 322.6 | 40 |
| ***50 nm*** | −49.41 | 0.0097 | 0 | 346.2 | 50 |

**Table S3:** Fitting parameter of the profiles extracted from the TEM images for Fe nanowires of 10 and 45 nm nominal thickness.

| | $t_{Nom}$ | $A$ (nm) | $C$ (nm$^{-1}$) | $y_c$ (nm) | $2y_0$ (nm) | $z_0$ (nm) |
|---|---|---|---|---|---|---|
| ***Full Profile*** | *10 nm* | -10.3(1) | 0.0213(7) | 0 | 263(1) | 12.45(8) |
| | *45 nm* | -47(2) | 0.0086(6) | 0 | 309(8) | 54(1) |
| ***Half Profile (left-side)*** | *10 nm* | -9.97(9) | 0.0225(6) | 0 | 257(1) | 12.69(7) |
| | *45 nm* | -48.8(6) | 0.0099(2) | 0 | 343(2) | 49.3(3) |

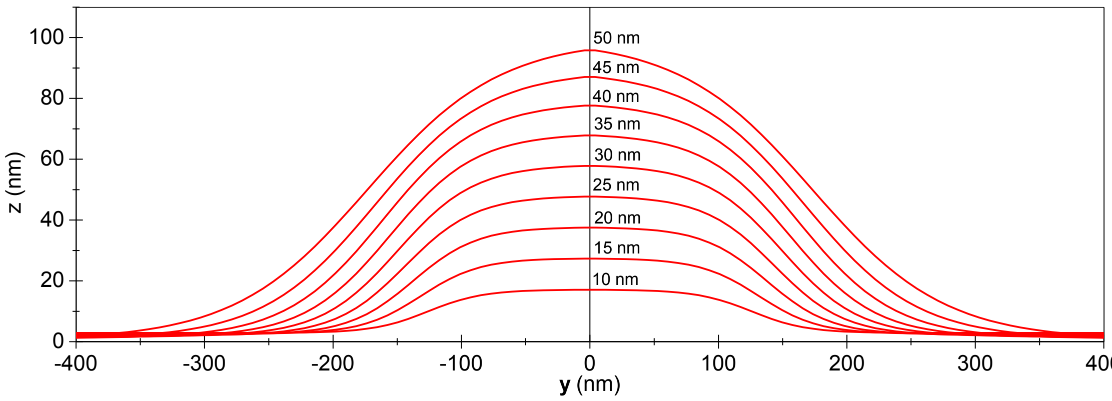


**Figure S2:** Simulated profiles using the Equation 2 in the main text and the fitting parameters reported in Table S2.
